# A Pilot Study Exploring the Relationship Between Milk Composition and Microbial Capacity in Breastfed Infants

**DOI:** 10.3390/nu17020338

**Published:** 2025-01-18

**Authors:** Ashwana D. Fricker, Kristija Sejane, Mina Desai, Michael W. Snyder, Luis Duran, Rachel Mackelprang, Lars Bode, Michael G. Ross, Gilberto E. Flores

**Affiliations:** 1 Department of Biology, California State University, Northridge, CA 91330, USA; ashwana.fricker@csun.edu (A.D.F.);; 2 Biology Department, Adelphi University, Garden City, NY 11530, USA; 3 Department of Pediatrics, Larsson-Rosenquist Foundation Mother-Milk-Infant Center of Research Excellence (MOMI CORE), and the Human Milk Institute (HMI), University of California San Diego, La Jolla, CA 92093, USAlbode@health.ucsd.edu (L.B.); 4 The Lundquist Institute at Harbor-UCLA Medical Center, 1124 West Carson Street, Torrance, CA 90502, USA; mdesai@lundquist.org; 5 Department of Obstetrics and Gynecology, David Geffen School of Medicine, University of California Los Angeles at Harbor-UCLA, Torrance, CA 90502, USA; 6 Department of Obstetrics and Gynecology, Charles R. Drew University, Los Angeles, CA 90059, USA

**Keywords:** human milk oligosaccharides, infant, microbiome, metagenome

## Abstract

Background: Maternal obesity may contribute to childhood obesity in a myriad of ways, including through alterations of the infant gut microbiome. For example, maternal obesity may contribute both directly by introducing a dysbiotic microbiome to the infant and indirectly through the altered composition of human milk that fuels the infant gut microbiome. In particular, indigestible human milk oligosaccharides (HMOs) are known to shape the composition of the infant gut microbiome. The goal of this study was to characterize the HMO profiles of normal-weight and overweight mothers and to quantitatively link HMO concentrations to the taxonomic composition and functional potential of the infant gut microbiome. Methods: Normal-weight (BMI = 18.5–24.9; *n* = 9) and overweight/obese (OW/OB; BMI > 25; *n* = 11) breastfeeding mothers and their infants were enrolled in this single-center, cross-sectional pilot study. Human milk from the mothers and rectal stool swabs from the infants were collected 7–9 weeks postpartum. The HMO composition, microbiome composition, and microbial functions were assessed using HPLC, 16S rRNA gene sequencing, and metagenomic sequencing, respectively. Results: Neither the HMO profiles nor the infant microbiome composition varied according to maternal BMI status. Taxonomically, the gut microbiota of infants were dominated by typical gut lineages including *Bifidobacterium*. Significant correlations between individual HMOs and bacterial genera were identified, including for *Prevotella*, a genus of the Bacteroidota phylum that was positively correlated with the concentrations of lacto-*N*-neotetraose (LNnT) and lacto-*N*-hexaose (LNH). Using metagenomic assembled genomes, we were also able to identify the broad HMO-degradative capacity across the *Bifidobacterium* and *Prevotella* genera. Conclusions: These results suggest that the maternal BMI status does not impact the HMO profiles of human milk. However, select HMOs were correlated with specific bacterial taxa, suggesting that the milk composition influences both the taxonomic composition and the functional capacity of the infant gut microbiome.

## 1. Introduction

The global obesity epidemic contributes to many negative health outcomes, including type 2 diabetes, cardiovascular disease, cancer, and early mortality. Although once a disease primarily affecting adults, the rates of childhood obesity are on the rise [1]. The pathogenesis of childhood obesity may begin in utero, where many studies have identified links between maternal obesity, high-fat diets, or gestational diabetes and infant and childhood obesity (reviewed in [2,3]). Notably, across four studies, maternal obesity was associated with a higher risk of offspring obesity (Odds Ratio, 3.06; 95% Confidence Interval, 2.68–3.49; *p* < 0.001), while numerous others have shown an increased risk of childhood obesity from the ages of 2–18 years [4,5,6,7,8,9,10].

The increased risk for infant and childhood obesity from having overweight mothers may be a result, in part, of a programmed hypothalamic development that results in increased orexigenic neurons, infant food intake, and excessive post-natal weight gain (reviewed in [11]). The increased appetite and decreased satiety in infants born to overweight mothers may be further exacerbated by higher caloric milk produced by overweight/obese (OW/OB) women [12]. Human milk consists of over 200 components, including carbohydrates (lactose and human milk oligosaccharides (HMOs)), lipids, proteins, and other bioactive compounds (hormones and antibodies) [13,14,15]. The concentrations of these components vary with many factors, including the time of day, stage of lactation, volume of milk produced, maternal diet, and maternal body mass index (BMI) [13,16].

Another potential contributing factor to childhood obesity is the human gut microbiome [17]. In adults, the human gut microbiome is recognized as an environmental risk factor that contributes to obesity [18,19]. Less is known about how the gut microbiome may be linked to childhood obesity, although both direct and indirect maternal effects during early life may be contributing factors. For example, the initial seeding of the neonatal microbiome occurs at birth with the delivery mode influencing the colonization and persistence of specific bacterial taxa [20]. Given that OW/OB mothers have an altered microbiome composition [21,22], the initial seeding of vaginally delivered infants could directly alter the composition and successional development of the gut microbiome [20,23]. Indirectly, the composition of human milk is known to be influenced by the maternal BMI [12,24,25]. In particular, the HMOs present in human milk, which are the primary growth substrates for members of the infant microbiome, have recently been shown to differ based on the maternal weight [25,26,27,28], and select, individual HMOs are predictive of infant weight gain [29]. While HMOs have numerous postulated health benefits to the developing infant [30], quantitative linkages between HMOs and infant microbiome compositions are not well characterized.

To begin to understand linkages between HMOs and features of the infant microbiome across maternal BMI statuses, we characterized the HMO composition of milk from 20 mothers and the taxonomic and functional composition of the gut microbiome of their 7–9-week-old infants. Given that maternal health influences the milk composition, we hypothesized that infants exclusively fed human milk from either normal-weight or OB/OW mothers would host taxonomically distinct, yet functionally redundant, microbial communities constrained by the milk HMO profiles. In this pilot study, we expanded upon studies comparing the infant gut microbiome to maternal milk [31,32] and identified a set of microorganisms with distinct genomic potential for the metabolism of specific HMOs.

## 2. Materials and Methods

### 2.1. Recruitment

Study Participants: This pilot study of twenty infant–mother pairs was a part of a previous study [12] approved by the Institutional Review Board at the Lundquist Institute at the Harbor-UCLA Medical Center (Study #18CR-32182). The enrollment and milk sample collection details were as described previously [12]. Briefly, women who delivered singleton, term neonates while inpatients at the Harbor UCLA Medical Center were recruited. All mothers were committed to exclusive breastfeeding during the first two months of the infant’s life. These women were grouped into two BMI categories, based on their pre-pregnancy BMI: normal-BMI women (18.5–24.9) and OW/OB women with a BMI ≥ 25. The demographics of the mother–infant pairs are summarized in Supplemental Table S1.

Study Visit: All studies were performed in the outpatient clinic of the Clinical and Translational Research Center (CTRC) between 10 a.m. and 12 p.m. to avoid potential circadian changes in the milk composition. Milk samples were obtained at least 1.5 h after the prior infant feed and mothers were asked to feed from one breast only in the feeding prior to the appointment. The opposite breast was then used for milk sampling. Continuous milk samples were obtained in 10 mL aliquots, and pumping continued until the primary breast was emptied or there was no further milk production.

Rectal Sample Collection: At the time of the milk collection, a rectal stool sample was collected from the infant using a rectal stool collection kit (a sterile, DNA-free, individually wrapped swab with nylon flocking and an ABS handle and a dry tube). A single rectal stool sample was collected from each infant by inserting the tip of the sterile swab beyond the anal sphincter by approximately 1 cm and rotating it to sample the anal crypts for 30 s. The swab was broken at the score line and placed in a dry tube with a screw cap and stored at −80 °C until thawing for DNA extraction.

Sample Size: A total of 9 women with a normal BMI and 11 women who were OW/OB were studied. Milk HMO analysis was undertaken on the foremilk (sample of the first 10 mL of milk collected) and hindmilk (last aliquot collected) samples. Rectal stool samples were obtained from 9 male (normal-BMI mothers, *n* = 6; OW/OB mothers, *n* = 3) and 11 female (normal-BMI mothers, *n* = 3; OW/OB mothers, *n* = 8) infants.

### 2.2. HMO Detection

The concentrations of nineteen major HMOs in milk were identified using high-performance liquid chromatography (HPLC) with fluorescence detection at the University of California, San Diego [33]. The HMOs that were quantified included 2′-fucosyllactose (2′-FL), 3-fucosyllactose (3-FL), 3′-sialylactose (3′-SL), 6′-sialylactose (6′-SL), lacto-*N*-tetraose (LNT), lacto-*N*-neotetraose (LNnT), difucosyllactose (DFLac), lacto-*N*-fucopentaose I (LNFPI), lacto-*N*-fucopentaose II (LNFPII), lacto-*N*-fucopentaose III (LNFPIII), sialyl-lacto-*N*-tetraose b (LSTb), sialyl-lacto-*N*-tetraose c (LSTc), difucosyllacto-*N*-tetraose (DFLNT), lacto-*N*-hexaose (LNH), disialyllacto-*N*-tetraose (DSLNT), fucosyllacto-*N*-hexaose (FLNH), difucosyllacto-*N*-hexaose (DFLNH), fucodisialyllacto-*N*-hexaose (FDSLNH), and disialyllacto-*N*-hexaose (DSLNH). The average concentrations of HMOs for each mother are shown in Supplemental Table S2.

### 2.3. DNA Extraction

DNA was extracted using the Qiagen PowerSoil Pro kit (Qiagen, Redwood City, CA, USA), using the manufacturer’s protocol with the following modifications. Swabs containing infant stools were cut directly into a PowerBead Pro Tube (Qiagen, Redwood City, CA) containing lysis beads using disinfected scissors. A sterile swab was processed and sequenced as a negative control. To lyse bacterial cells, the PowerBead Pro Tubes with the swabs were placed on a horizontal vortex adapter at max speed for 10 min. After elution with a 50 μL elution buffer, the DNA concentrations were read on a Qubit 2.0 fluorometer with a high-sensitivity kit (Invitrogen, Life Technologies Corporation, Carlsbad, CA, USA), following the manufacturer’s protocol. DNA was stored at −20 °C until thawing for 16S rRNA gene amplification, qPCR, or metagenomic sequencing.

### 2.4. 16S rRNA Gene Sequencing and Analysis

To characterize the composition of each infant microbiome, the V4 region of the 16S rRNA gene was PCR-amplified with the barcoding primer set 515F/806R ([34]; “16S Illumina Amplicon Protocol: Earth Microbiome”, n.d.) as previously described [35,36].

Sequences were analyzed following the QIIME-2 Atacama Desert pipeline. Briefly, sequences were denoised, dereplicated, and chimeras were removed with Dada-2 [37] using the following parameters: --p-trim-left 5 and --p-trunc-len 150. Amplicon sequence variants (ASVs) were classified using sklearn and the silva 138.99 database [38]. Extraction blanks (containing no swabs) were used to remove contaminating ASVs following the decontamR pipeline [39] using a prevalence-based strategy with the default probability threshold (0.1). Mitochondrial and chloroplast sequences were also removed. QIIME-2 was used to calculate various α-diversity (the total ASV counts and Shannon Diversity Index) and β-diversity (Bray–Curtis and Jaccard) metrics using a sampling depth of 70,000 sequences per sample.

### 2.5. Enumeration of Bifidobacterium Using Quantitative PCR (qPCR)

Because *Bifidobacterium* are known primary colonizers of the gut of breastfed infants [40,41], we determined their absolute abundance using quantitative PCR (qPCR). The standard curve was generated from the near-full-length 16S rRNA gene amplified from the genomic DNA of *Bifidobacterium longum* spp. infantis ATCC 15697 using the 8F and 1492R primers [42] following established protocols [43]. Quantitative PCR (qPCR) using a BioRad CFX96 Real-Time System (Bio-Rad Laboratories, Hercules, CA, USA) was used to determine the number of 16S rRNA gene copies per ng of DNA extracted. The final 20 μL reaction mixture consisted of 1X GoTaq qPCR reagent (Promega, Madison, WI, USA), 0.5 μM primers Bif-F and Bif-R [44], and 1 μL of each sample. The protocol included an initial polymerase activation step at 95 °C for 5 min, followed by 34 cycles of denaturation at 95 °C for 15 s, annealing at 58 °C for 20 s, and extension at 84 °C for 30 s with a plate read; after 5 min of a final extension at 72 °C, a melt curve was implemented from 65 °C to 95 °C in increments of 0.5 °C every 10 s. The reactions were performed in triplicate on each plate and each sample was run three times in independent qPCR reactions.

### 2.6. Metagenome Sequencing and Analysis

To characterize the functional potential of each infant’s gut microbiome, genomic DNA extracted from rectal stool swabs was sent to SeqCenter (SeqCenter LLC, Pittsburg, PA, USA) for metagenomic sequencing. Metagenomic libraries were prepared using an Illumina DNA prep kit and a target size of 320 bp. Reads were sequenced on an Illumina NovaSeq 6000 sequencer (Illumina, San Diego, CA, USA), producing 2 × 151 bp paired-end reads. The read statistics are shown in Supplemental Table S3.

The metagenome sequences were analyzed following previously established workflows [45,46]. Briefly, sequences were trimmed with Trimmomatic-0.39 with the following parameters: LEADING: 3, TRAILING: 3, SLIDINGWINDOW: 4:15, and MINLEN: 36. Following trimming, human sequences were removed with BBMap-39.01, by aligning the sequences to a masked HG19 genome [45] using the minid = 0.95 parameter.

### 2.7. Metagenome-Assembled Genome (MAG)-Based Analysis

Each clean, human-decontaminated infant metagenome read set was assembled using SPAdes (v3.11.1 with --meta flag) [47] and then checked with MetaQuast.v3.2 [48]. The assembly statistics are reported in Supplemental Table S4. After mapping reads with Bowtie2 (v2.5.0) [49,50], contigs greater than 200 bp were binned using Metabat2 (v 2.15) [51], and bins were refined using Magpurify (v2.1.2) [52]. High- and medium-quality MAGs (<10% contamination and ≥50% completeness) as determined by CheckM (v1.2.2) [53] were selected for further analysis based on Bowers, 2017 [54]. The MAG taxonomy was predicted using GTDBTK (v2.1.1) [55,56], CDS regions predicted using Prodigal (v2.1.2) [57], and predicted genes annotated using HMMER (v3.3.2) (hmmer.org) with the dbCan (v12.0) database, downloaded in October 2023 [58,59]. The raw contig abundance and coverage results from Bowtie were used to calculate the normalized per-MAG mean and standard deviation abundance and coverage statistics. For normalization, the total number of reads in a sample (specific fastq pair) was first divided by one million, then the mapped reads were divided by the total number of sequences and finally divided by the total contig length (in kb) to obtain the reads per kb per million mapped reads (RPKM). High-quality MAG taxonomy and quality statistics are reported in Supplemental Table S5.

### 2.8. Statistical Analysis

All statistical tests were computed and the figures were generated using R 4.1.1 (R Foundation for Statistical Computing, Vienna, Austria). For all diversity metrics, the Shapiro test was used to determine normality using either base R or the rstatix package (rstatix v.0.7.2 [60]). Comparisons were considered significant if the corrected *p*-values were less than 0.05 except where indicated. Symbols in the figures show nonsignificant (ns), 0.05 (*), 0.01 (**), 0.001(***), and <0.0001(****) *p*-values. The Rmarkdown files used to calculate statistics and generate figures are available at https://github.com/ashfricker/baby-microbes (accessed on 13 January 2025).

Milk: For the fore- and hindmilk α-diversity analyses, significant differences in the paired samples were calculated using a paired Wilcoxon test with FDR corrections (observed HMOs, individual HMOs) using the rstatix package or a paired *t*-test (Shannon). For the β-diversity comparisons of the fore- and hindmilk, Euclidean distance matrices were generated and statistically significant differences were calculated using the mantel test from the vegan package (vegan v.2.6-6.1 [61]) or multiple regression on distance matrices (MRMs) from the ecodist package (ecodist v.2.1.3 [62]). To determine differences in the HMO composition across maternal BMI categories, fore-and hindmilk were averaged and differences in α-diversity were determined using a Wilcoxon test. Significant differences in the β-diversity were determined using a PERMANOVA based on Euclidean distance matrices.

Microbiome: For the β-diversity of the microbial taxonomies, dissimilarity matrices generated in Qiime-2 were used to calculate statistically significant differences with ADONIS from the vegan package. Comparisons were considered significant if the *p*-value of the F statistic was less than 0.01. The Shapiro test in base R was used to determine normality, followed by a *t*-test or Wilcoxon test to determine if the maternal BMI category and infant sex were associated with the microbiota α-diversity (the total ASV counts and Shannon Diversity Index) or MAG abundances (RPKM) using the rstatix package.

Combined: Microbiome features (e.g., the subset ASVs or MAG RPKM) were compared with the HMO composition using Spearman’s rank correlation coefficient using the cor.test function in base R. Due to the exploratory nature of the analysis, *p*-values were not corrected.

## 3. Results

### 3.1. Participant Characteristics

As part of a previous study aimed at understanding the relationship between the human milk composition (fat and calorie content) and blood serum composition (lipids and insulin) within the context of the maternal BMI, 20 mother–infant dyads provided fore- and hindmilk samples and infant rectal swabs at 7–9 weeks postpartum. The demographic and health information for the study population is summarized in Supplemental Table S1, where the milk fat content is derived from the foremilk sample.

### 3.2. HMO Composition

First, to identify whether the HMO composition shifted between fore- and hindmilk samples, the summed and individual HMO distributions, α-diversity (the observed HMOs, Shannon Evenness, and Inverse Simpson), and β-diversity (the Euclidean distances across samples) were measured and statistically tested for differences. The averaged HMO concentrations in the fore- and hindmilk are shown in Supplemental Table S2.

The HMO concentrations did not vary between the fore- and hindmilk samples when comparing either individual HMOs or HMO concentrations summed by the type (sialylated or fucosyllated) (Supplemental Figure S1). Similarly, across all α-diversity metrics, no significant differences were identified between the fore- and hindmilk even when selecting for secretor mothers only (Supplemental Figure S2). In line with the α-diversity results, the distance matrices of the fore- and hindmilk HMO composition for all mothers or secretor mothers only were not significantly different, suggesting similarity across these fractions. Therefore, subsequent analyses used the average of both the fore- and hindmilk.

**Figure 1:**
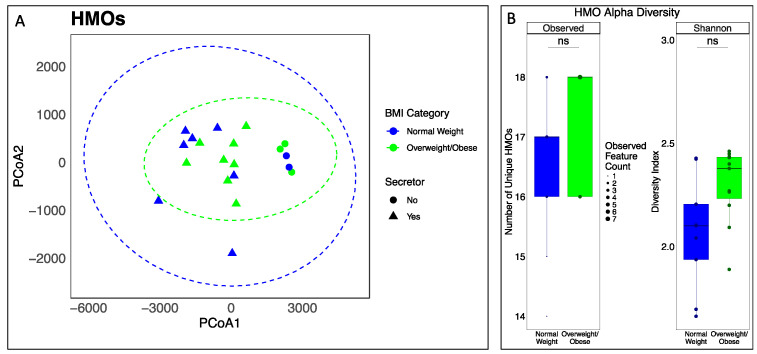
The human milk oligosaccharide (HMO) profiles of mothers were not different across the pre-pregnancy BMI statuses. Principle coordinates plot of the Euclidean distance of all HMOs measured using HPLC across all mother–infant pairs (**A**) and the α-diversity metrics of HMOs according to the maternal BMI status (**B**). For the β-diversity metrics, a PERMANOVA indicated that the HMO profiles across maternal BMI status were not statistically significant (*p* > 0.05). For the α-diversity metrics, statistically significant differences were calculated using a Wilcoxon test (ns = nonsignificant).

To determine whether the HMO composition might by influenced by the maternal weight, mothers were grouped by their BMI status, and both α- and β-diversity metrics were compared (Figure 1). Both a PERMANOVA based on the HMO composition (β-diversity) and comparisons of the HMO richness and evenness (α-diversity) indicated no significant differences (*p* > 0.05, R^2^ = 0.06) across milk samples from normal-weight versus OW/OB mothers, even when selecting for secretor mothers (*p* > 0.05, R^2^ = 0.15). Comparing the concentrations of individual HMOs between normal-weight and OW/OB mothers also revealed no significant associations, even when selecting for secretor mothers only (*p* > 0.05) (Supplemental Figure S1).

### 3.3. Infant Stool Microbiome Composition and Diversity

**Figure 2:**
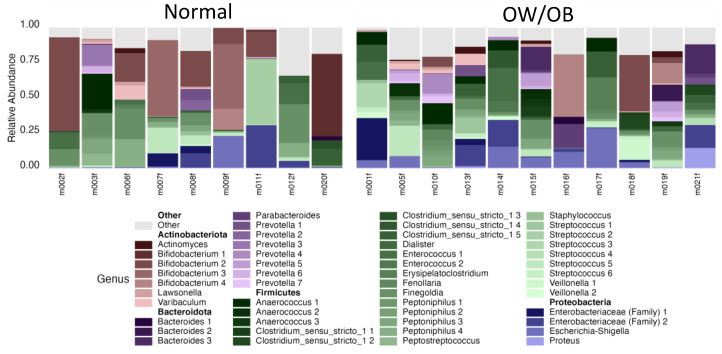
Communities from infants birthed by normal-weight (BMI = 18.5–24.9) or overweight/obese (OW/OB, BMI > 25) mothers were dominated by Actinobacteriota and Firmicutes. Each color shade represents a distinct taxonomic group that has been labeled down to the genus, when possible, with numbers representing different species within the genus. The dominant color represents a phylum: Actinobacteriota (brown), Bacteroidota (purple), Firmicutes (green), and Proteobacteria (blue). The top forty taxa are shown, while remaining low-abundance OTUs are grouped in Other (light gray).

The sequencing of the 16S rRNA gene from the 20 infant gut samples resulted in a total of 2,499,573 sequences. Samples were denoised, dereplicated, and then chimeric sequences, contaminating sequences based on extraction blanks, and mitochondrial sequences were removed, resulting in 86.7% of the original sequence total (2,166,578 sequences) being used for analysis. Taxonomic assignment of sequences across all infants identified Actinobacteria (Actinobacteriota), Bacteroidetes (Bacteroidota), Firmicutes (Bacillota), and Proteobacteria (Pseudomonadota) as the dominant phyla (Figure 2). Of these, Actinobacteria, Firmicutes, and Proteobacteria were present in all infants, whereas Bacteroidetes were only observed in 18 infants. However, the abundance of these phyla varied widely, ranging from 0.6% to 73% (Actinobacteria), 0.4% to 79% (Firmicutes), and 0.4% to 36% (Proteobacteria). Similarly, when present in infants, the abundance of Bacteroidetes ranged from 0.01% to 33% of the total sequence reads.

At the genus level, *Streptococcus* and *Finegoldia*, members of the Firmicutes, were found in all infants, with other genera of this phylum present in most infants (Figure 2). Of the Actinobacteriota, *Bifidobacterium* was present in most infants (*n* = 19) and had the greatest abundance of any single genus (up to 72.81%), with *Actinomyces* and *Lawsonella* also prevalent. Although the Bacteroidetes were not present in all infants, two genera of this phylum, *Prevotella* and *Bacteroides*, were represented in over half the infants, ranging from abundances of 0.01% to 26.3% and 0.01% to 23.91%, respectively.

**Figure 3:**
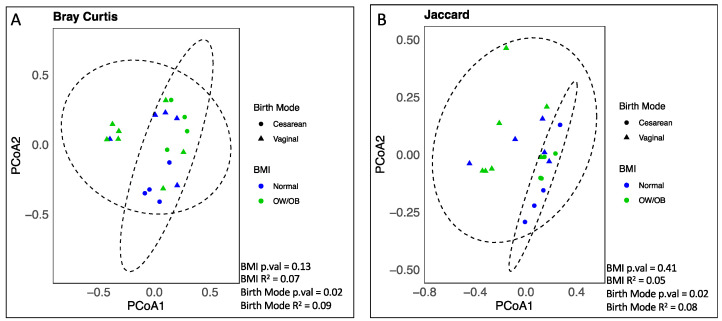
Microbial community structures were unique across birth modes but not maternal weight statuses. Principal coordinates analysis of community structures associated with maternal weight and delivery mode, as determined by Bray–Curtis (**A**) or Jaccard (**B**) metrics using 16S rRNA gene amplicon sequencing. Ellipses represent 95% confidence level for multivariate t-distribution based on infant birth mode. PERMANOVA calculated in R indicated that microbial communities across maternal BMIs were not significantly different, but microbial communities were statistically different based on birth mode.

To determine whether the maternal BMI status significantly impacted the infant gut microbial β-diversity, we performed a PERMANOVA of Bray–Curtis dissimilarities across the two groups. The gut microbiome of infants from normal-weight mothers was not significantly different than in those from OW/OB mothers (PERMANOVA *p*-value > 0.05, R^2^ = 0.07). Next, to identify the potential alternate stratification of the study population, we compared the β-diversity across other known characteristics of the mothers and infants, such as the delivery mode, secretor status, ethnicity, and sex. The only significant association was with the delivery mode (PERMANOVA *p*-value < 0.05, R^2^ = 0.09) (Figure 3), which was also observed using an unweighted metric (Jaccard, Figure 3B).

**Figure 4:**
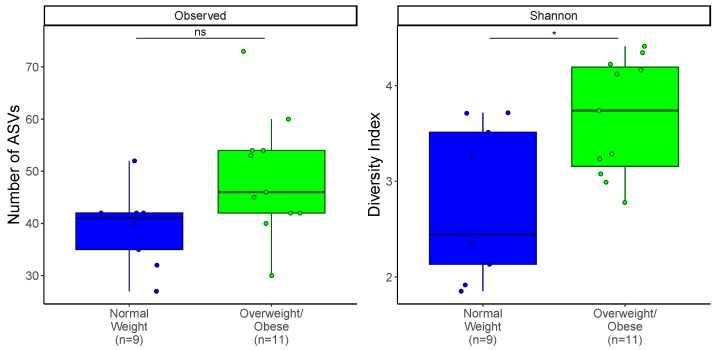
Infants from normal-weight mothers have lower gut microbial evenness than infants from overweight/obese (OW/OB) mothers. Alpha diversity analysis of infant gut community membership for infants from normal-weight (BMI = 18.5–24.9) or overweight and obese (BMI > 25) mothers. Mean and first and third quartiles of number of ASVs and Shannon Evenness are presented. Statistically significant differences were calculated using *t*-test; *p* > 0.05 (ns), *p* < 0.05 (*).

To identify a possible effect of obesity on the community richness and evenness (α-diversity), both the total ASV counts and Shannon entropy were analyzed. Both richness and evenness of the gut microbial communities of infants from OW/OB mothers were higher compared to those of infants from normal-weight mothers, although this difference was only significant for the evenness (*t*-test *p* < 0.05, Figure 4). Across all other categorical variables, the only other significant difference was a higher richness in female infants (Supplemental Figure S3).

We next examined the distribution of the most abundant phyla and genera in relation to the BMI. When comparing taxa from infants birthed by normal-weight mothers to those birthed by OW/OB mothers, none of the phyla were associated with maternal BMI (Maaslin qval > 0.25; NB: this is the default qval significance for Maaslin2). Within the Actinobacteria, the genus Bifidobacterium had the greatest frequency of occurrence (present in 19 infants), but the overall abundances between the infants of normal-weight or OW/OB mothers were not significantly different (qval > 0.25, Supplemental Figure S4A). To confirm these results, qPCR analysis with *Bifidobacterium*-specific primers similarly indicated no significant difference in *Bifidobacterium* abundances between groups (Supplemental Figure S4B). Notably, however, the species of Bifidobacterium were evolutionarily closely related across infants when present (Supplemental Figure S5).

### 3.4. Microbial Associations with HMOs

**Figure 5:**
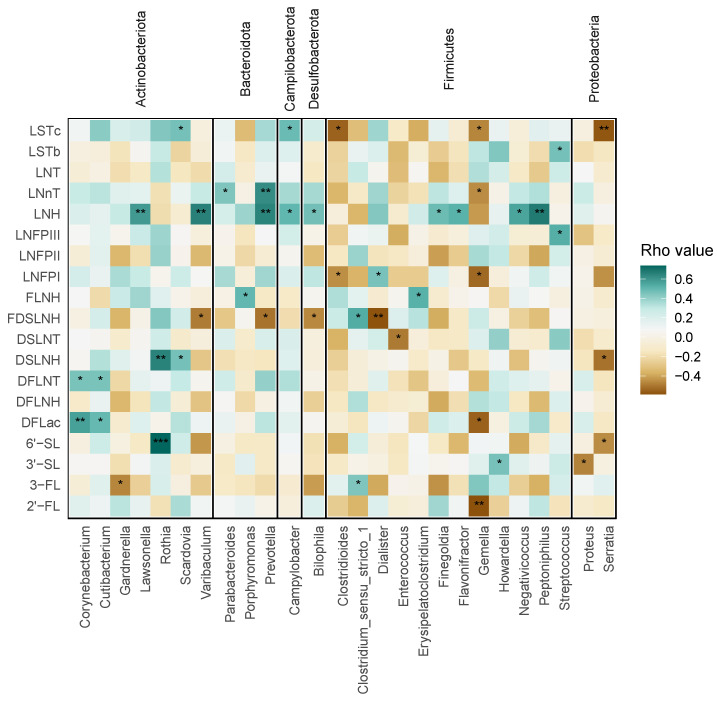
Bacterial taxonomic groups significantly associated with at least one HMO. Spearman rank correlation coefficients were calculated for all taxa and HMOs detected. The heatmap color reflects the direction and intensity of the correlation, with significant interactions indicated by asterisks; uncorrected *p*-values are represented by symbols: 0.05 (*), 0.01 (**), <0.001 (***).

The relatedness of infant Bifidobacterium and the absence of this genus in some samples suggested potential HMO metabolism by non-bifidobacterial species. To address this possibility, bacterial genera were compared to the HMO abundances using Spearman rank correlations (Figure 5), which revealed significant positive correlations between Rothia, a member of the Actinobacteria, and both 6′SL and DSLNH. Other genera of Actinobacteria with positive associations included Corynebacterium and Cutibacterium with both DFLac and DFLNT. Similarly, one genus belonging to Bacteroidetes, Prevotella, was positively associated with two HMOs, LNnT and LNH. Surprisingly, no organisms were positively associated with 2′FL, the most abundant HMO produced by secretor mothers, although Gemella, a member of the Firmicutes, was negatively associated with this HMO.

### 3.5. Infant Stool MAGs

To identify genes that may have been involved in HMO catabolism or related to the maternal weight status, microbial DNA was submitted for metagenomic sequencing, resulting in a total of 168,967,258 sequences. After quality control, sequence reads mapping to human DNA were removed. The final number of sequences analyzed constituted 47% of the original sequence total (79,463,822 sequences).

To identify which taxa may have been involved in HMO catabolism, metagenomic reads were assembled and binned into MAGs. A curated list of 152 medium- and high-quality MAGs was used for all downstream analyses (Supplemental Table S5). At the domain level, all of the MAGs were annotated as belonging to Bacteria. At the phylum level, a high number of MAGs were annotated as belonging to Firmicutes (43%), followed closely by Actinobacteriota (32%), Bacteroidota (15%), and Proteobacteria (9%). Firmicutes were predominately represented by members of the *Streptococcaceae* and *Enterococcaceae* families at proportions of 58% and 29% of Firmicutes, respectively. Organisms that likely were involved in HMO metabolism included members of the Actinobacteriota phylum, whose MAGs were identified as *Bifidobacteriaceae* (38%), *Actinomycetaceae* (38%), and *Mycobacteriaceae* (13%). Other potential HMO-degrading organisms included the phylum Bacteroidetes, which was dominated by the *Bacteroidaceae* (87%) and *Tannerellaceae* (13%) families.

To determine whether specific bacterial families were associated with the maternal BMI, the relative abundance of MAGs was used. None of the families were associated with the BMI (Supplemental Figure S6), but *Bifidobacteriaceae* had slightly higher RPKM values in normal-weight mothers as compared to overweight and obese mothers, in line with 16S rRNA gene sequence findings. Associations with HMOs irrespective of the maternal weight category were identified though Spearman rank correlations (Supplemental Figure S7). Similarly to observations made with the 16S rRNA gene sequence analysis, the family *Bacteroidaceae* was associated with LNnT. In addition, this family was associated with DFLNT and the low-abundance LNH. Other families belonging to the phylum Actinobacteriota were associated with multiple HMOs, including a positive association between *Mycobacteriaceae* and LNH and a negative association between *Actinomycetaceae* and LSTc.

**Figure 6:**
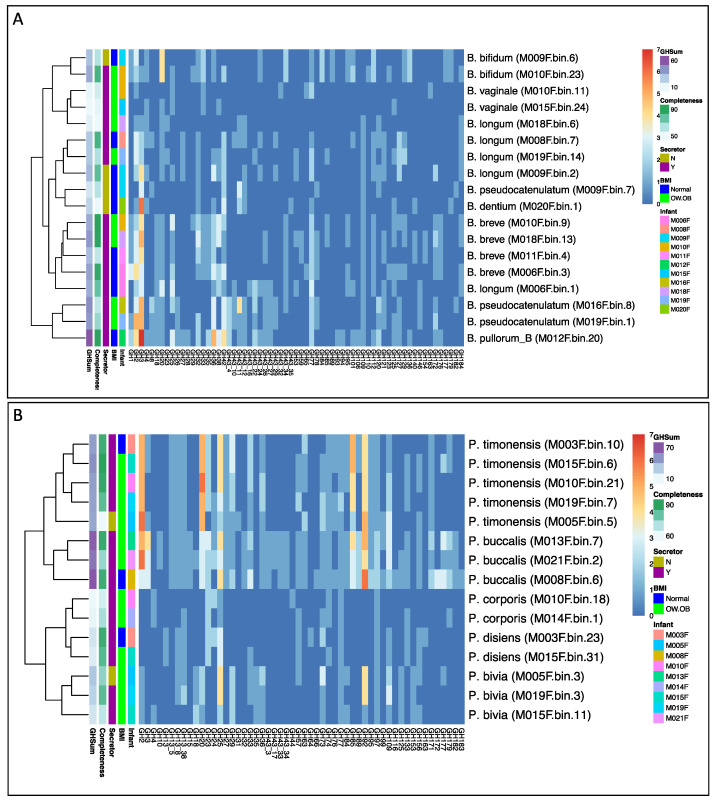
Putative glycoside hydrolase genes abundant in high- and medium-quality MAGs from Bifidobacterium (**A**) and Prevotella (**B**) indicate potential HMO-degrading capabilities across related MAGs. Column annotations include infant sample (Infant), maternal weight status (BMI), maternal secretor status (Secretor), MAG completeness (Completeness), and total GH copies (GHSum). Taxonomy was assigned using GTDB-Tk (Supplemental Table S5). MAGs are clustered by Euclidean distances.

To identify which taxa retained enzymes relevant to HMO catabolism, the richness estimates of GHs in the MAGs averaged across each phylum were compared (Supplemental Figure S8). In general, taxa that had more negative associations with HMOs, such as members of the Firmicutes, contained a lower number of GH families (average of 10) compared to taxa that had positive associations with HMOs, including Actinobacteriota (average of 15) and Bacteroidota (average of 36). Within the Actinobacteriota, 18 MAGs from 11 infants were classified as belonging to *Bifidobacterium*, including *B. vaginale*, which is classified as *Gardnerella vaginalis* in the NCBI taxonomy database. Of the members of Bifidobacterium, there was a high variation in the copy number of GH2 and GH3 genes (Figure 6A). Similarly, but perhaps expectedly given its correlation with HMOs in this population, the MAGs classified as *Prevotella* (15 MAGs across 9 infants) also generally clustered by the GH copy number, varying greatly in the number of gene copies of GH2 and GH20, enzymes involved in the catabolism of LNnT (Figure 6B).

## 4. Discussion

Children born to overweight mothers are at an increased risk of developing childhood obesity [4,5,6,7,8,9,10]. To begin to understand if mother’s milk plays a role in this predisposition through alterations of the infant gut microbiome, we attempted to link differences in HMO composition of the mother’s milk across BMI categories with the taxonomic and functional potential of their infant’s microbiome. In this population, we did not find any significant associations between the maternal BMI status and overall HMO content of milk. Likewise, we did not find strong associations between the maternal BMI category and infant microbiome composition, potentially due to a small survey population, although these results are in line with similar studies in infants [32]. Taxonomically, most infants hosted *Bifidobacterium*, and while the abundances were higher in infants from normal-weight mothers, these associations were not statistically significant. Despite the absence of significant associations between the overall HMO content and microbiome composition, we found strong associations between select bacterial taxa and individual HMOs. For example, one taxon positively associated with select HMO abundances with the genomic potential to deconstruct HMOs is *Prevotella*. While *Prevotella* species have been observed in other infant cohorts [40,41], this is the first report linking them to HMO abundance, providing intriguing directions for future studies.

A previous study with this same population of mothers found that overweight and obese individuals produced milk with a higher caloric and fat content than milk from normal-weight mothers [12]. We did not see the same patterns with respect to HMOs and literature searches on this relationship yielded conflicting results. For example, a negative relationship between the maternal BMI and LNnT has been observed [27], while other studies have shown a positive correlation between the pre-pregnancy BMI and LNnT [25,26], and yet others have demonstrated no correlation between the maternal BMI and LNnT [32]. These differences across studies comparing the HMO composition with the maternal BMI could be due in part to the sampling time, where differences in the HMO composition have been observed over the lactation time [26,63]. Overall, this suggests that obesity-associated metabolic alterations may not influence HMO synthesis pathways. From a practical standpoint, this means that higher fat and caloric contents [12], leptin loads [64], adenine levels [65], or mannose concentrations [24] combined with higher intakes due to decreases in the satiety of infants [12] are more likely to influence infant obesity than milk oligosaccharides. Notably, changes in hormone levels including those of leptin and ghrelin in mothers may lead to hyperphagia, leptin resistance, and weight gain in offspring [66,67,68]. It is also possible that the microbiota is responding to other milk components, including milk fat composition, milk intake, vitamin levels, hormone abundances [68], or immune factors that may vary from person to person [15]. Future studies could address the influence of other milk components on the infant gut microbiota.

In the prior study [12], the milk fat content increased 4-fold from the foremilk to hindmilk samples. In the present study, we demonstrated a remarkable similarity in the HMO composition between foremilk and hindmilk samples. These results indicate that the metabolic processes that result in milk fat are independent of those producing HMOs. Therefore, studies of the milk HMO composition can likely utilize foremilk, hindmilk, or pooled samples to assess representative HMOs. However, it remains uncertain whether the HMO composition may vary by the breast, time of day, or weeks postpartum.

One of the more novel results from this study is the association between select HMOs and the abundance of *Prevotella*. While *Prevotella* are common inhabitants of the oral, vaginal, and gut microbiomes of adults, they have not previously been studied in the context of HMO degradation. In the gut, *Prevotella* are typically more abundant in adults who consume high-fiber and low-fat diets [69,70,71] and increase in abundance during the transition from milk to solid foods [72] or after the cessation of HMO interventions [73]. Some *Prevotella* have been shown to degrade other host-produced glycans including mucin [74,75] and vaginal glycocalyx [76,77,78] using a suite of glycoside hydrolase enzymes [75,78]. Their potential to degrade HMOs may be expected given that other gut bacteria co-opt mucin-degrading enzymatic machinery to degrade HMOs [79], and further exploration of this capacity is warranted.

While this report has several strengths, there are some limitations. First, we only included a single sampling point of 20 mother–infant dyads, limiting stratification by other factors including the mode of delivery, infant sex, secretor status, and time. While this report included metagenomic sequencing of infant rectal stool samples, which enabled the interrogation of functional differences, the investigation of effects associated with maternal diet, disease, developmental stage, mode of delivery, generational species transfers, and infant health status will need a prospective study design using a larger, balanced cohort of mother–infant pairs. Here, we found that organisms in the infant gut have a capacity for increased energy harvesting but did not evaluate the potential for these organisms to have an increased production of metabolites that contribute to obesity. Characterizing the metabolic output by analyzing fecal short-chain fatty acids, metabolites implicated in immune function and cellular energy [80], would enable the mapping of genomic features onto metabolic models and deepen our understanding of the potential for the infant microbiome to contribute to childhood obesity.

## 5. Conclusions

In conclusion, although the maternal BMI status has been identified as a risk factor for childhood obesity, these data suggest it may not be mediated through the HMO composition at this stage of life. Nevertheless, across all mothers, select HMOs were correlated with specific bacterial taxonomic groups, suggesting the potential to use specific HMOs to modulate the gut microbiome composition. We would urge caution in overinterpreting these results as we only present a single snapshot of the microbiome/milk composition, and we do not know any health outcomes for the infants surveyed. These data would be aided by a large, balanced longitudinal study that tracked the infant health status, including weight gain alongside the milk composition and consumption, and the infant gut microbial composition and metabolic output, which would reveal key metabolites involved in the long-term health effects of gut microbiome development. The potential use of individual HMOs in these longitudinal studies to track infant outcomes is a starting point to develop strategies for the rational design of therapies to modulate the infant gut towards host health.

## Data Availability

All raw sequencing data in a FASTQ format are available in the NCBI Sequence Read Archive (SRA) BioProject database under the accession number PRJNA1138764 as BioSamples SAMN42742152–SAMN42742171.

## Supplementary Materials

The following supporting information can be downloaded at: https://www.mdpi.com/article/10.3390/nu17020338/s1, Table S1: Mother-Infant Dyad Demographics, Table S2: Human Milk Oligosaccharide (HMO) profile of mothers at 7–9 weeks after delivery as measured by HPLC, Table S3: Number of sequences after trimming and mapping to the human genome used in downstream analyses, Table S4: Assembly metrics of contigs determined by Quast, Table S5: Medium and High-quality MAG quality and taxonomy statistics, Figure S1: Human Milk Oligosaccharide (HMO) profile of fore and hind milk is statistically the same across all mothers, Figure S2: Human Milk Oligosaccharide (HMO) α-diversity of fore and hind milk is statistically the same across all mothers and secretor mothers only, Figure S3: Female infants have higher microbial richness than male infants, Figure S4: Bifidobacterium are not associated with maternal BMI status, Figure S5: Closely related Bifidobacterium are present in half of the infants, Figure S6: No taxa identified in the high-medium quality MAGs were associated with maternal BMI status, Figure S7: The abundance of the Bacteroidaceae family is associated with multiple HMOs, Figure S8: The phyla Bacteroidota and Proteobacteria encode for the greatest diversity of GHs.
