# Supplementary figures and images for: A Pilot Study Exploring the Relationship Between Milk Composition and Microbial Capacity in Breastfed Infants
Source: Nutrients. 2025 Jan 18;17(2):338. doi: 10.3390/nu17020338 (PMC11768495; doi:10.3390/nu17020338)

Supp Figure S1

A

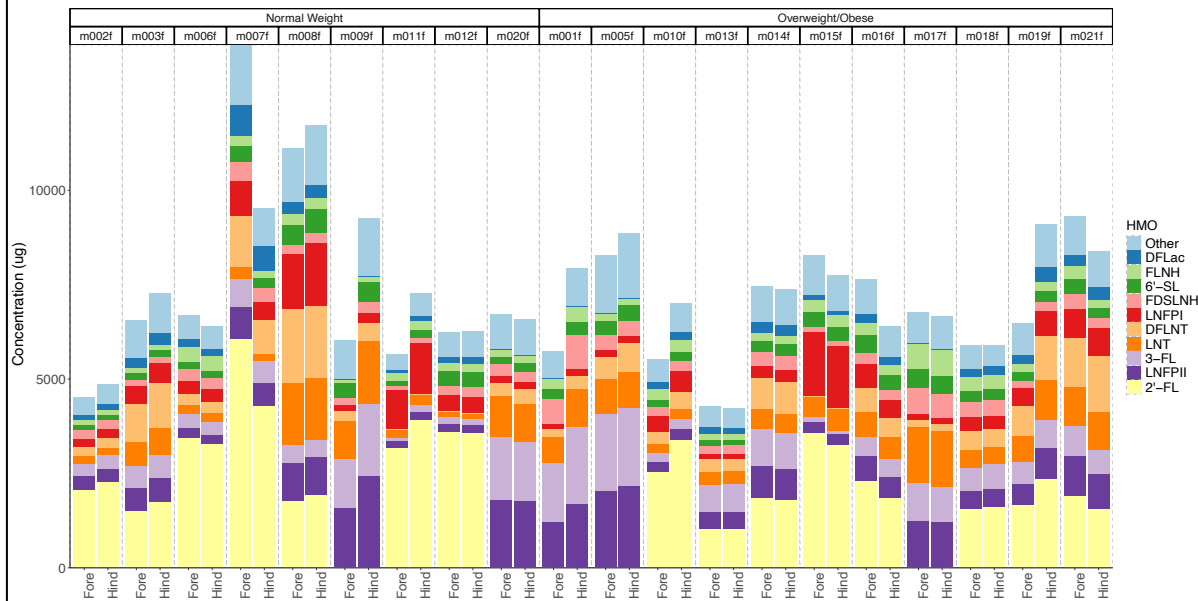

B

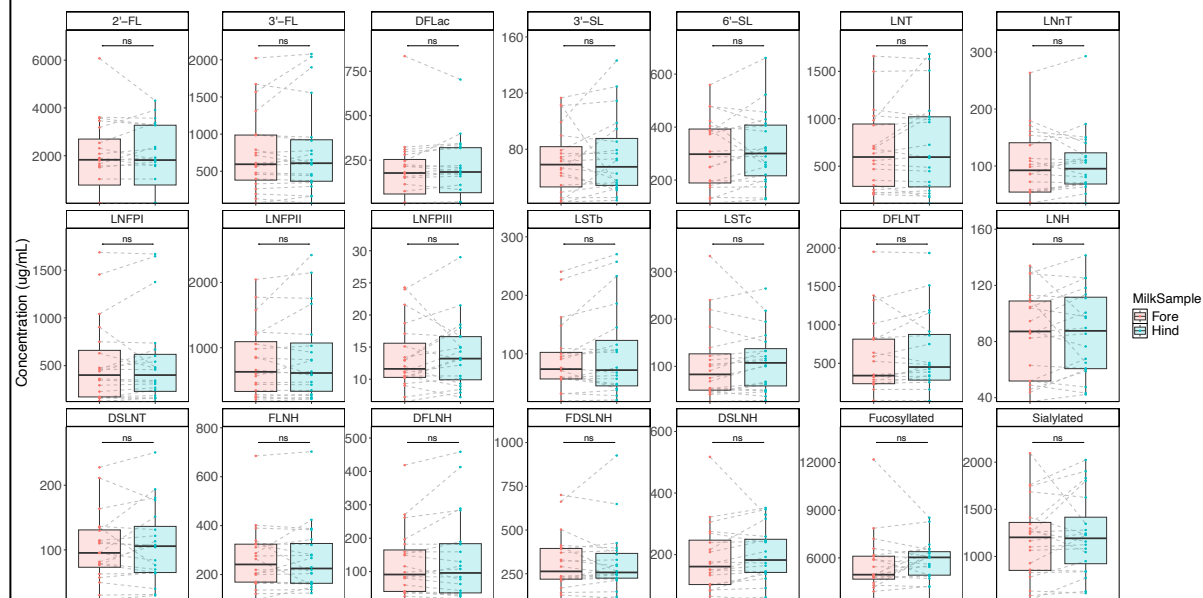

Supplement: Supplementary file 1 [file nutrients-17-00338-s001.zip › Supp Figure S1.pdf]

# Supp Figure S2

## A

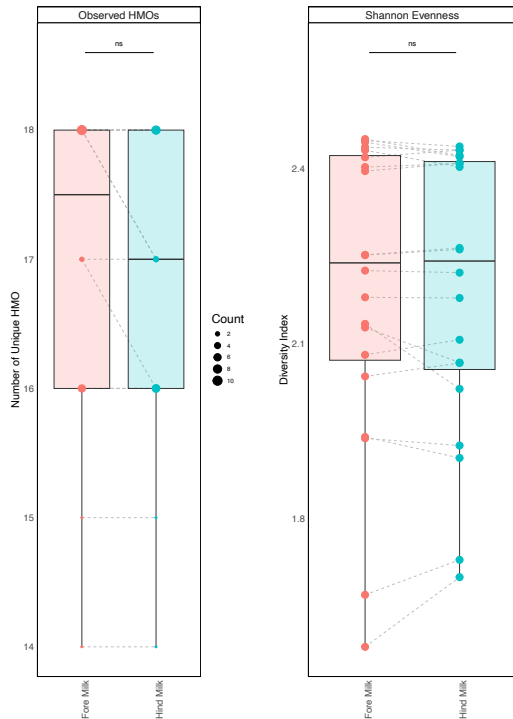

## B

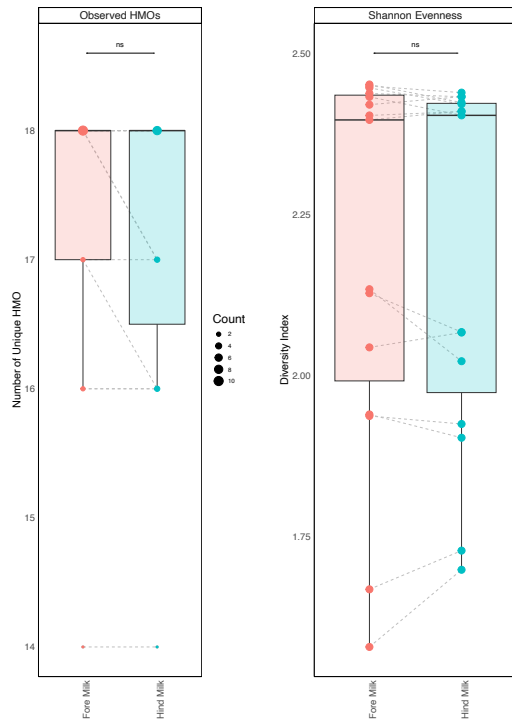

Supplement: Supplementary file 1 [file nutrients-17-00338-s001.zip › Supp Figure S2.pdf]

Supp Figure S3

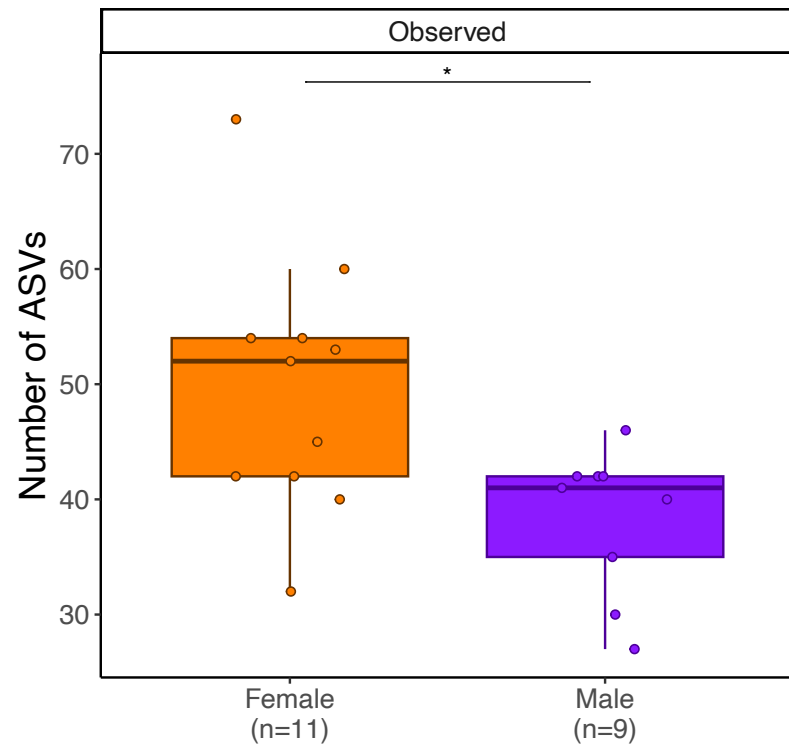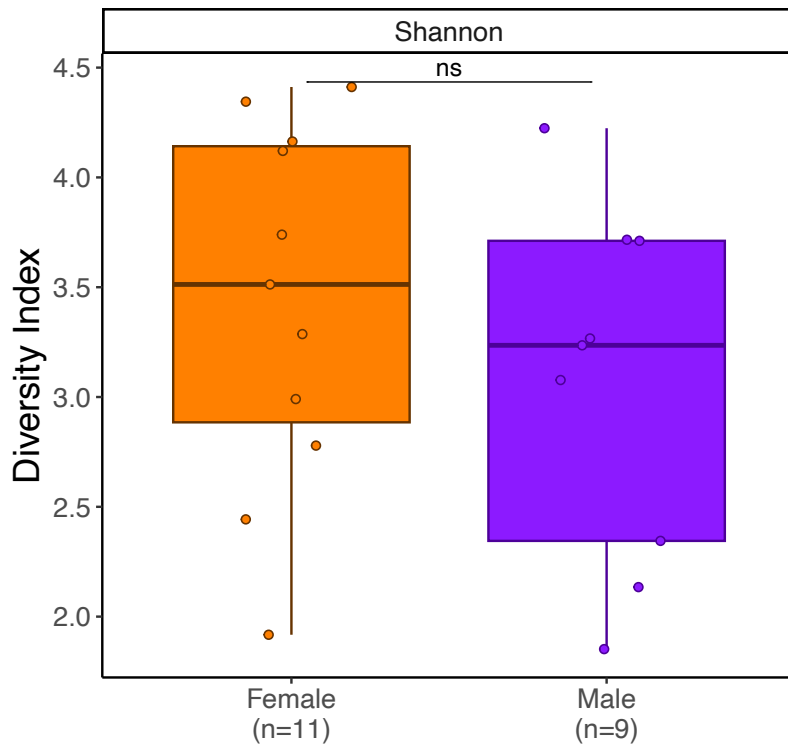

Supplement: Supplementary file 1 [file nutrients-17-00338-s001.zip › Supp Figure S3.pdf]

Supp Figure S4

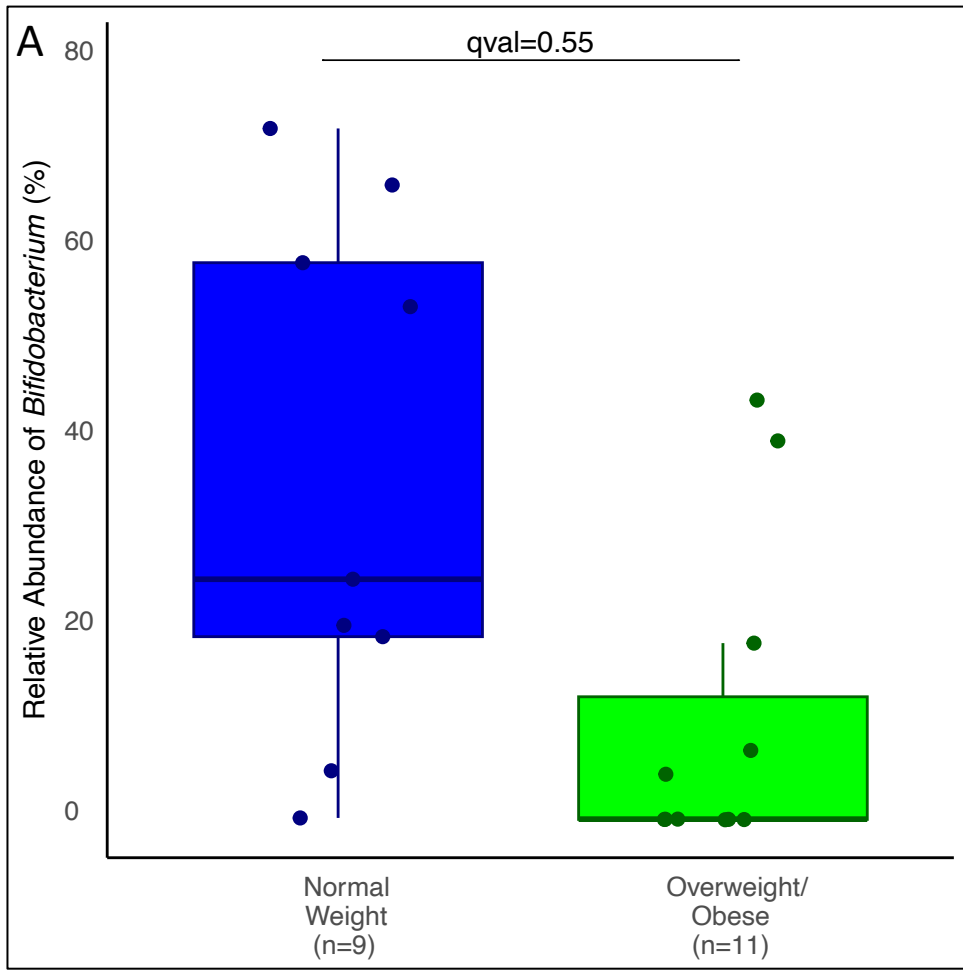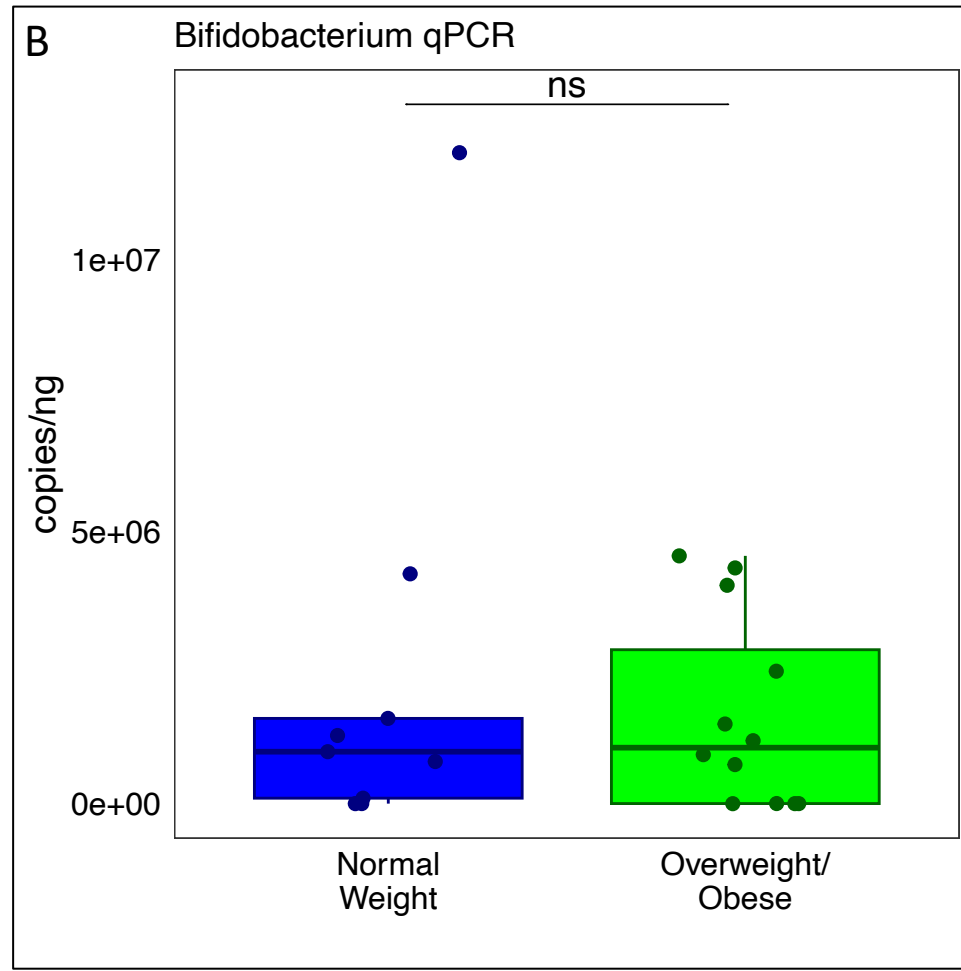

Supplement: Supplementary file 1 [file nutrients-17-00338-s001.zip › Supp Figure S4.pdf]

# Supp Figure S6

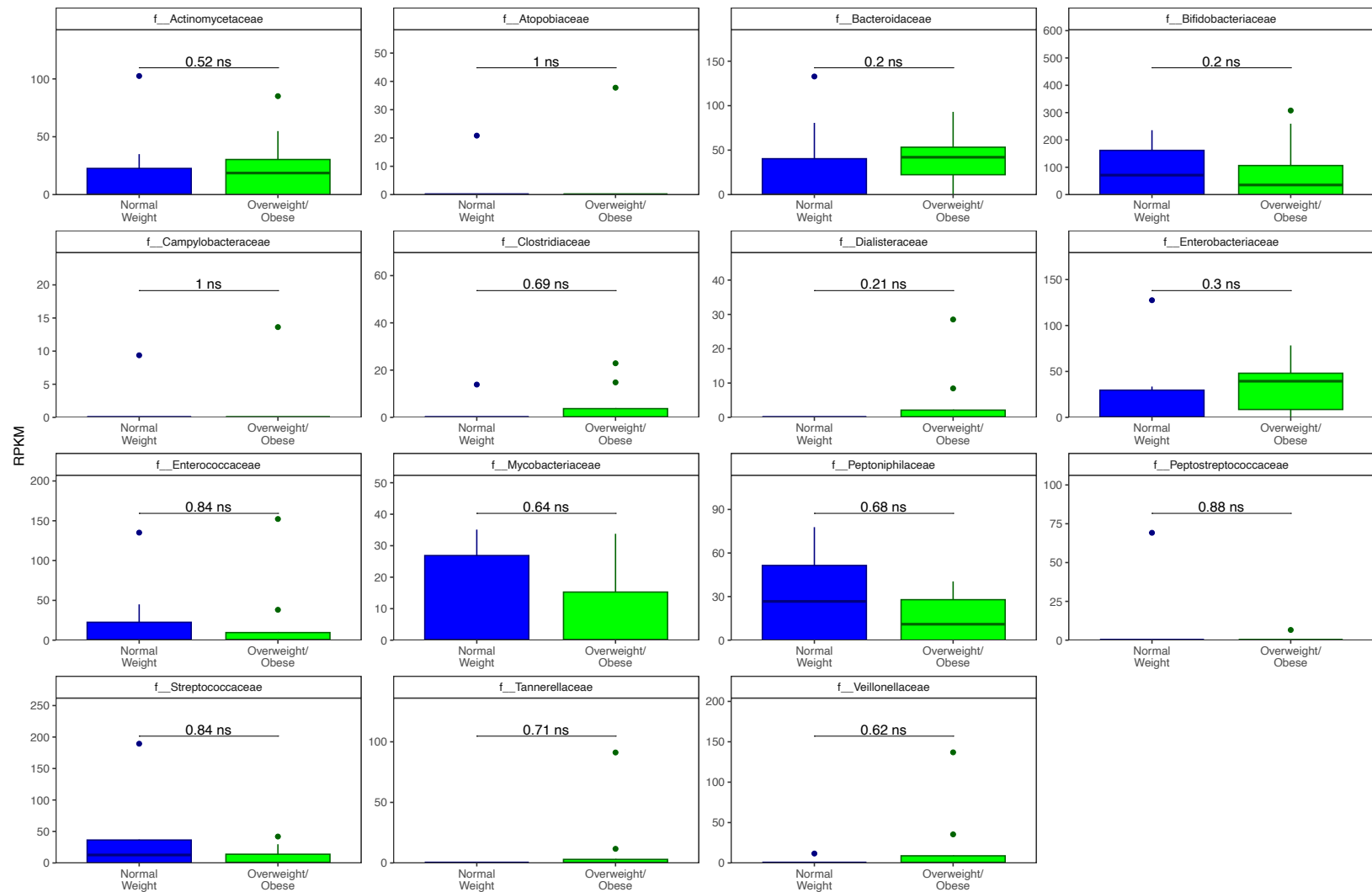

Supplement: Supplementary file 1 [file nutrients-17-00338-s001.zip › Supp Figure S6.pdf]

Supp Figure S7

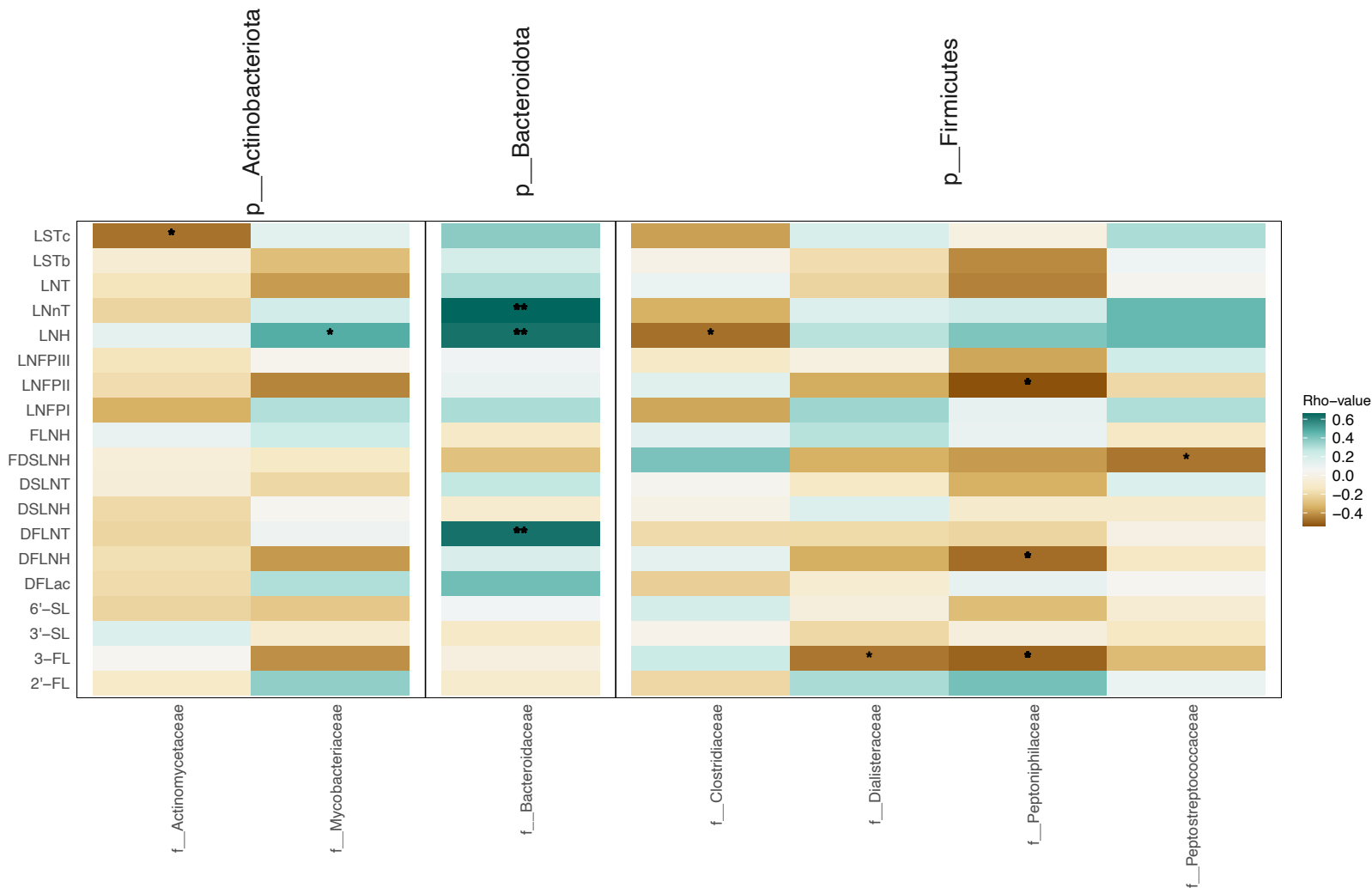

Supplement: Supplementary file 1 [file nutrients-17-00338-s001.zip › Supp Figure S7.pdf]

Supp Figure S8

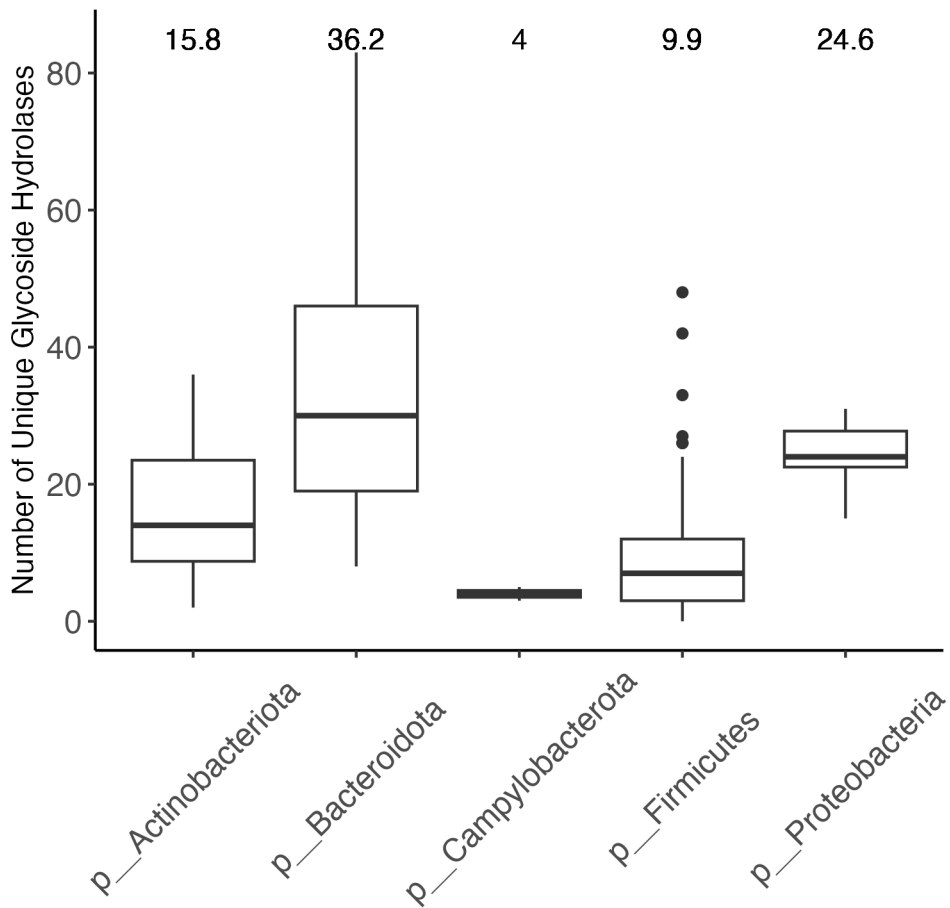

Supplement: Supplementary file 1 [file nutrients-17-00338-s001.zip › Supp Figure S8.pdf]
