# Supplementary material for: A Pilot Study Exploring the Relationship Between Milk Composition and Microbial Capacity in Breastfed Infants
Source: Nutrients. 2025 Jan 18;17(2):338. doi: 10.3390/nu17020338 (PMC11768495; doi:10.3390/nu17020338)

Supp Figure S5

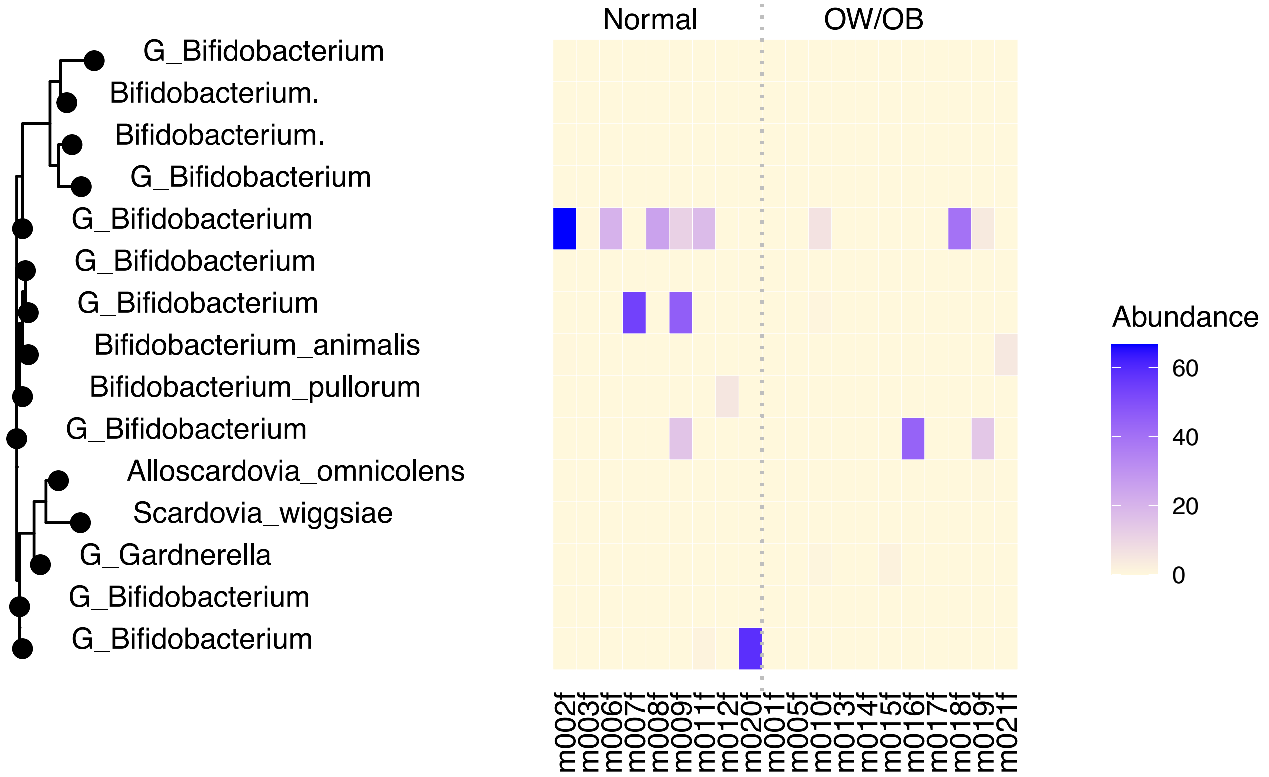

Supplement: Supplementary file 1 [file Supp Figure S5.pdf › Supp Figure S5.pdf]
